# Remarks on the President’s Address

**Published:** 1909-02

**Authors:** John C. Olmsted

**Affiliations:** Atlanta, Ga.


### REMARKS ON THE PRESIDENT'S ADDRESS.

BY DR. JOHN C. OLMSTED, ATLANTA, GA.

*Mr. President and Gentlemen*:

The admirable address which we have just heard, presents so many important subjects for our consideration, and contains such valuable suggestions, as to our future action, that I find myself embarrassed, in selecting an especial topic, with which to open the discussion. I shall, however, begin with one, in which I have had some personal experience, as a member of the Board of Censors of this Society.

The President referred to the relations of the profession to the public press of this city; especially in the matter of newspaper notices, of members of our profession, in which the "operations" of certain physicians were exploited, and their connection with the cases of certain "prominent citizens" was heralded forth; I may say in gross violation of ethical propriety, and proper professional dignity—not seldom indeed, has the public been favored with the pictures of these distinquished gentlemen, in order, I suppose, that there might be no possible mistake as to their identity.

The President urged that we relax not our efforts to suppress this unprofessional "advertising," for it is nothing else, in the eyes of every right-minded, high-toned physician. Indeed, gentlemen, I believe that we are a scandal to the profession of the state, in this respect. Unless I am greatly mistaken, no such misconduct as this, exists, or would be tolerated, by the profession in Savannah, Augusta and Macon, which I mention, as being of Georgia's larger cities. Our President suggests that the influence of this society be brought to bear upon the editors of our daily papers, in explaining our position in this matter, and endeavoring to have them eliminate the names of physicians, in connection with "news items," of the character mentioned. I think this effort should be made, or rather continued; for it has already been made; and the President will readily recall his experience with Dr. E. C. Davis and myself, when as members of the Board of Censors, we called as a "Committee from the Fulton County Medical Society," several years ago, upon the editors of the three daily papers of this city. I may say that we were courtously received, and our statements listened to with patient attention; but I think we shall not soon forget, the amusing smile, that gradually suffused the faces of two of the more genial of them; a smile as of one pitying the ignorance of innocence! And then these gentlemen told us a few things. They mentioned how they were at times pressed by certain physicians, to put in these notices. "Why," said one of them, evidently putting himself under some restraint, "I reckon that you would be amazed to know the names of some of these doctors; they are among the most prominent of the profession." Another editor informed us that their "medical items" were often furnished by one of their "news centres;" further explained that the chief "news centre" of his paper, were "the courts," "police department," "the jail" and "the hospitals," as regards local matters. In the case of one "hospital," its claims to recognition were so frequent and persistent, the editor said, that he had given orders that no notice be taken of its "news items," etc. We were enlightened in regard to some matters, which we had gravely surmised, but not positively known. Two of the editors expressed a willingness to carry out the wishes of this society, as far as possible, without conflicting with their funstion as purveyors of "news" to public. The third, was rather non-committal: viz: "If my paper doesn't publish such matters in which the public is interested, another will," etc. I believe, however, that good would come, if this society presses this matter further, by formal action, and transmitting its sentiments, in the form of a request, to the editors of these papers. It is indeed humiliating, that we have to invoke such aid, in protecting the ethics of the profession, from infraction by its own members; yet only those of us, perhaps, who have occupied the unhappy position of membership on the "Board of Censors," can appreciate the difficulties, and embarrassments encountered, when endeavoring to investigate, and exercise our duties, in such cases of infraction.

The ignorance claimed as to "how such a notice got into the paper" is truly pathetic; as is also the negation of all knowledge as to "how they got hold of my picture!"

There are, it would seem, among the insoluble mysteries of our present state of existence! All of this too, in the face of certain specific data, minutive of detail, sometimes even biographical of the doctor, which would plainly indicate the source as being the innocent victim himself. Of course, we are aware of cases, or rather instances, in which a physician has been made the victim of well-meaning, but mistaken friends, who only meant to do him a kindness, by giving him a "good send off" before the public; but this only shows the effect upon the public mind, of the demoralizing results of the advertising members of our profession, in degrading their noble calling, to a commercial level.

Let me devoutly express the hope, that as regards the Board of Censors, and this class of offenses, that this new year will be one of blessed calm and freedom!

A few words on another topic, and I will not further try your patience.

The President has rightly suggested our duty to the public, in regard to taking some measures to enlighten, and protect them, as concerns the abounding quackery in this city. Georgia has long been the "dumping ground" of unscrupulous medical quacks; and Atlanta, if she is not "the State of Georgia" (as is claimed by some) is certainly the centre of this industry.

We should not be discouraged by past failures in this direction. I recall some of these. It sometimes seems as if the public did not wish to be "protected" from these quacks, and that the saying of P. T. Barnum, "the American people love to be hum-bugged," is especially appliable in this instance.

The regular profession is presumed to be jealous of the "Indian remedy," the negro's recipe, and the wonderful concoction of "Old Dr. Gridly!" I recall the case of a notorious quack whom we prosecuted some years ago. We had the most conclusive evidence, complying with all the technical requirements of the law; the judge's charge pleased us, but so did not the jury's verdict, which acquitted the rascal, promptly. As I have quoted Barnum's classic remark, I will quote another classic one as expressive of my feelings on this accasion; it was that of the late Commodore Vanderbilt; "the people may be d—d!" However, this sort of philosophy won't do; we must be more patient, and philanthropic.

But sir, if we are to accomplish results in this matter, whether in municipal or state legislation, it must be by the co-operation of the three recognizedly legal medical systems of this state, Regular, Eclective and Homeapathic. These three professions stand equal before the law, and their harmonious and combined action, in all medical legislation must be obtained, if effectual warfare on the common enemy of illegal practitioners is to be waged. They have co-operated with us in the past, they will do so, I am sure in the future, and are capable of wielding a powerful influence upon public opinion, for we must remember that with many of the public, their influence is greater than our own.

But, I have already occupied too much of your time, and will only say in conclusion, that I trust all your efforts in the varied fields before us, will result in the upholding of the best traditions of a noble profession, in an age of commercialism, and recall to our benefit, as remarked by our President, those high ideals of an older day, which, whatever were its short-comings, caused the title of "Doctor" to be respected.

